# Cell Death Pathways and Phthalocyanine as an Efficient Agent for Photodynamic Cancer Therapy

**DOI:** 10.3390/ijms160510228

**Published:** 2015-05-06

**Authors:** Ivan Mfouo-Tynga, Heidi Abrahamse

**Affiliations:** Laser Research Centre, Faculty of Health Sciences, University of Johannesburg, P.O. Box 17011, Doornfontein 2028, South Africa; E-Mail: 200504361@student.uj.ac.za

**Keywords:** death mechanisms, photodynamic cancer therapy, reactive oxygen species, photosensitizer, phthalocyanine

## Abstract

The mechanisms of cell death can be predetermined (programmed) or not and categorized into apoptotic, autophagic and necrotic pathways. The process of Hayflick limits completes the execution of death-related mechanisms. Reactive oxygen species (ROS) are associated with oxidative stress and subsequent cytodamage by oxidizing and degrading cell components. ROS are also involved in immune responses, where they stabilize and activate both hypoxia-inducible factors and phagocytic effectors. ROS production and presence enhance cytodamage and photodynamic-induced cell death. Photodynamic cancer therapy (PDT) uses non-toxic chemotherapeutic agents, photosensitizer (PS), to initiate a light-dependent and ROS-related cell death. Phthalocyanines (PCs) are third generation and stable PSs with improved photochemical abilities. They are effective inducers of cell death in various neoplastic models. The metallated PCs localize in critical cellular organelles and are better inducers of cell death than other previous generation PSs as they favor mainly apoptotic cell death events.

## 1. Introduction

At the cellular level, death is a well-understood biological process and refers to the inability of a cell to preserve indispensable life functions. In mammalian cells, the process is important for both development and homeostasis [1]. Cell death assists life by removing damaged cells and superfluous cells that are capable of causing harm and, therefore, need to be destroyed for the advantage of the whole organism [2]. After suffering damages beyond repair and becoming inadequate for the entire organism, damaged cells undergo cell death mechanisms. For an individual to reach its post-embryonic maturity, superfluous cells, such as those in the interdigital spaces, are to be destroyed to prevent syndactyly [3]. An average individual loses half of his/her body mass annually, and without this process, the individual would have accumulated a bony mass of more than two tons and a 16 kilometer long intestine by the age of 80 [4].

### 1.1. Apoptosis

Depending on cellular aspects such as morphology, enzymatic activity, functional and immunologic responses, the mode of cell death is determined and designated as either a programmed or non-programmed mode [5]. Nuclear and membrane degradation characterize apoptosis, which is the standard programmed and also the most occurring cell death [6]. It is triggered by precise signals that lead to the activation of cascade pathways to finally deliver a suicidal response. Therefore, apoptosis is an induced and regulated process that activates a family of proteins (known as caspase) and precise cellular events, which degrade nucleic and polypeptide materials. Caspases are cysteine aspartyl proteases and can further stimulate others effector agents to digest cellular contents. Additionally, the apoptotic response is enhanced upon the binding of BH3-only members of the Bcl-2 family to and inhibiting the action of pro-survival proteins.

As a consequence, affected cells round up and cease communication with adjacent cells; their plasma membranes bleb, and phosphatidylserines translocate to the outer layers (Table 1). These mechanisms are accompanied by additional cellular changes, including cross-linkage and polymerization of proteins, chromatin condensation, nuclear fragmentation from ±300,000 to 185 nucleotides through internucleosomal degradation by cation-dependent endonuclease and, finally, cellular fragmentation into apoptotic bodies and removal. Permeable mitochondrial membranes and the release of apoptogenic substances, such as cytochrome C, characterize the intrinsic pathway, while the activation of death receptors, such as tumor necrosis factor receptor 1 (TNFR1) or Fas/CD95, on the plasma membrane characterizes the extrinsic pathway [3,6,7,8]. In contrast, the presence of Beclin 1 prevents caspase-dependent cell death events and demonstrates anti-apoptotic potential [8]. Silicon phthalocyanine (PC-4) photosensitizer (PS) was used in photodynamic therapy (PDT) to cause damage to CD4+ CD7− malignant T-lymphocytes. PC-4 was efficient at inducing cytodamage by destroying BCL-2 proteins and promoting apoptosis [9]. Similarly, a metallo-PC-mediated PDT in breast cancer cells led to apoptosis signs, including the predominance of apoptotic cells post-PDT; nuclear fragmentation was seen as oligonucleosomal degradation and increased expression of the B-cell lymphoma 2 (*Bcl-2*), DNA fragmentation factor alpha (*DFFA1*) and caspase 2 (*CASP2*) genes [10]. PSs that preferentially accumulate in mitochondria and damage BCL-2 protein, like PC, generally are inducers of apoptosis [11]. Additionally, the level of calcium ions (Ca^2+^) and the transfer from endoplasmic reticulum to mitochondria represent further criteria for cell death induction. The overload of Ca^2+^ in mitochondria leads to changes in morphology and apoptosis in a nonnuclear and Ca^2+^-dependent manner [12]. In the presence of an increased level of intracellular Ca^2+^, PDT has the ability to induce cytodamage, which also appears to be p53 dependent [13].

**Table 1:** Distinctive characteristics of cell death pathways. Different cell death pathways can be classified according to morphological appearance (apoptotic, autophagic, necrotic), enzymological criteria or regulators (distinctive classes of proteases, such as caspases, calpains and kinases) and functional aspects (programmed or accidental, physiological or pathological).

Distinctive Features	Cell Death Pathways		
	Apoptosis	Autophagy	Necrosis
Morphologies	Shrinkage; blebbing; chromatin condensation; DNA degradation; nuclear fragmentation, apoptotic bodies	Decreased cell size; double membrane vesicles; organelle degradation	Cell swelling; loss of membrane integrity; organelle swelling; NO DNA laddering
Regulators	Death receptors; Bcl-2 family; Beclin 1; caspases; IAPs; adaptor proteins; kinases; phosphatases; calcium ions, calpains; BCNI1	mTOR; PI3 kinase; ATG family; UPR stress sensors; Beclin 1; kinase (JNK); Bcl-2 family; IP3 receptor	Calcium ions; ion channels; metabolic failure; PARB, calcium-regulated proteins; RIP kinase; death receptors; ceramides
Stimuli	ROS; DNA damage; death receptors ligands; developmental programs; organelle stress; anti-cancer drugs; ER calcium release	Nutrient starvation; protein aggregation; ER stress; calcium overload; developmental programs; hypoxia; ischemia; damaged organelles; proteasome impairment	bacterial toxins; metabolic poisons; ischemia; stroke; calcium overload
Response	Programmed, physiological	Survival, accidental, physiological	Accidental, pathological

Abbreviations: ATG, autophagy; Bcl-2, B-cell lymphoma 2; IAPs, inhibitor of apoptosis proteins; IP3 receptor, inositol 1,4,5-trisphosphate (IP3) receptors; ER, endoplasmic reticulum; mTOR, (mammalian) target of rapamycin; PAR, poly(ADP-ribose); NO, nitrite oxide; PARB, PAR-binding site; PI3 kinase, phosphatidylinositide 3-kinases; UPR, unfolded protein response; ROS, reactive oxygen species; RIP1, oxygen species; RIP1, a specific kinase that is recruited to the death-inducing signaling complex.

### 1.2. Autophagy

Embryological studies revealed that cells are accumulated and damaged in vesicles during a programmed cell death known as autophagy, an essential, but selective cell degrading process [14]. Autophagy is thought to be primarily a pro-surviving mechanism initiated by cells that have to face sub-lethal levels of damage [15]. Additionally, autophagy was shown to promote the induction of immunogenic cell death and to play a critical role in photodynamic-related cell damages, especially in apoptosis-resistant cells [16]. When inhibiting autophagic actions, cancer cells showed resistance and increased cell survival after treatment [17]. Thirty autophagy-related genes (*Atg*) and two ubiquitin-dependent mechanisms have been identified, but Atg7 protein is involved in both mechanisms and participates in other Atg activations, autophagosomal assemblage and molecular degradation [18]. Unlike apoptosis, autophagy is not well understood, but has also been reported to be involved in either cell survival or death function [19].

Autophagy is a catabolic process comprised of reactive oxygen species (ROS) accumulation, membrane lipid oxidation on the membrane and loss of plasma membrane integrity in the absence of caspase activities. Caspase is a family of cysteine proteases that are inducers and effectors of changes occurring during apoptosis [3,20,21]. Caspase inhibition induces catalase degradation and ROS accumulation. The accumulation of ROS is dependent on the activity of catalase and is related to autophagy [22]. This mode of cell death has several roles in many biological pathways and differs from endocytosis lysosomal destruction by the formation of an autophagosome, which engulfs the target components to be degraded within the lysosomes [23]. Four consecutive steps make up this process, and these are: appropriation, transportation to lysosomes, degradation and reutilization of residues (Table 1). Lysosomes are the sites for cellular degradation and prompt cell death through the release of lysosomal hydrolases into the cytoplasm, prompting cell death. The action of autophagic proteins protects neighboring cells from the detrimental effects of lysosomal degradation [24]. A regulated autophagy controls the cell metabolism by degrading, recycling and synthesizing cell components [25,26]. PDT is recognized to activate apoptosis, but it can also induce autophagy in several types of cells, where the autophagic response can either involve survival or cell death mechanisms. Autophagy is first of all a survival mechanism, and autophagy-triggered PDT can add resistance to the therapy by inhibiting cell death signals. However, apoptosis-deficient cells depend on autophagic responses to induce cell death after PC-4 PDT. Both apoptosis and autophagy are required for an enhanced cell death response, and when blocking autophagy, MCF-7 cells develop resistance to PC-mediated PDT, resulting in increased cell survival [27,28]. PCs that localize in the endoplasmic reticulum and affect both the mammalian target of rapamycin (mTOR) activation and Beclin-1 protein are likely to lead to a comprehensively autophagic cell death response [11].

### 1.3. Necrosis

Necrosis has been referred to as an accidental and non-programmed cell death event. There is an absence of signals associated with programmed cell death, and an inflammatory response characterizes this mode of death. External stimuli, such as infections, toxins and trauma, are required to initiate a necrotic cell death response [5,29]. In the absence of caspase activity in eukaryotic cells, it was shown that signal transduction and catabolic activities govern the execution of the necrotic pathway through the death domain and toll-like receptors. The activity of the receptor interacting protein (RIP1) controls the promotion of this cell death. RIP1 is a serine/threonine kinase required for the death receptor signaling and necroptosis, which is a necrotic-like and caspase-independent programmed cell death (Table 1) [30]. Seven types of necrotic cell death pathways have been recognized, but the sequential events remain unchanged for all. They include membrane permeability, movement of calcium ions across the endoplasmic reticulum, cytoplasmic swelling (oncosis), calcium-dependent calpain activation, lysosomal rupture, followed by degradation of cell components and induction of the inflammatory response [31]. Though it lacks the distinctive characteristics of apoptosis and autophagy, necrosis is comprised of precise sequential events; therefore, it can occur in a controlled manner. The efficiency of the liposomal aluminum chloro-phthalocyanine (AlClPC) in PDT was studied both *in vitro* and *in vivo* using the oral cancer cell line Ehrlich tumor cells, and AlClPC-mediated PDT led to 90% necrotic cell death and disruption of blood vessels [32,33]. Another PC, zinc phthalocyanine tetra-sulfonated, was found to possess anti-neoplastic activity after PDT, and an evident increase of necrotic-related cell death was seen [34]. Subcellular localizations with cell membrane disintegration, local depletion of oxygen and nutrients are prone to receive PCs that would stimulate cell death by necrosis [11].

The relative uptake, cytodamage and subcellular localization are all dependent on distinctive chemical features of each PC. Neutral PCs showed more diffuse localization and were likely to primarily localize in the Golgi apparatus in the perinuclear area. Though both cationic and anionic PCs prefer lysosomes as their initial sites of localization, the cationic, followed by the neutral PCs appeared to be more effective than their anionic counters. Following irradiation, PCs undergo relocalization, which is charge dependent, and this demonstrated that the secondary localization site is more important in predicting the outcome of any PC-mediated PDT [35].

### 1.4. Reactive Oxygen Species and Photodynamic Cancer Therapy

ROS have been considered only as a metabolic by-product and are intentionally produced by the Nox family NADPH oxidases on cell membranes during immune response. These phagocyte oxidases are crucial in innate immunity and are usually inactivated in resting cells. During phagocytosis, they are activated to produce ROS, which are the precursors of oxidants. This generation of ROS during phagocytosis makes Nox family NADPH oxidases major effectors in the protection mechanism [36,37]. In mitochondria, ROS stabilize hypoxia-inducible factors (HIF-1), which are a transcriptional regulator of the immune response and are essential for the secretion of tumor necrotic factor (TNF-alpha) [38]. The action of ROS on HIF-1 changes the mitochondrial functions and leads to the modulation of the immune response. This altered immune function can enhance lifetime [39]. Patients with dysfunctional oxidases suffer from improved vulnerability to microbial infection. ROS attack bacteria in the isolated neutrophil phagosomes. Nox family NADPH oxidases and ROS are important in innate immunity for the eradication of microbial infection [40].

Elevated levels of ROS, downregulation of ROS scavengers and antioxidant enzymes are associated with various human diseases, including diabetes, neurodegenerative diseases and various cancers. Cancer is a multistage disorder and a leading cause of death worldwide. The term cancer is characterized by the proliferation and invasion of abnormal cells without control in an organ. The abnormal cells can spread to other organs and body locations through the blood or lymph systems. Several types of cancer have been identified, and the most-commonly diagnosed include breast cancer, colorectal cancer, endometrial cancer, kidney cancer, lung cancer, cervical cancer, skin cancer, non-Hodgkin lymphoma, melanoma, leukemia, pancreatic cancer, prostate cancer and thyroid cancer [41,42]. Early diagnosis is critical for the efficiency of the treatment. Surgery, radiation therapy and chemotherapy are effectively used in the battle against cancer, but their lack of specificity for cancer cells causes damage to normal healthy cells, numerous side effects and loss of cell functions. Thus, there is an increasing interest to develop more sensible and effective discriminatory means of treating cancer [10,43].

**Figure 1:**
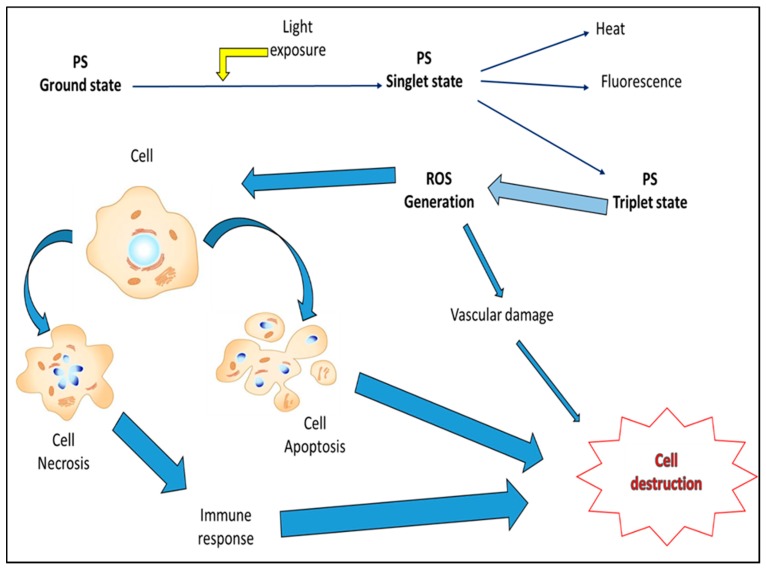
Photodynamic cancer therapy (PDT)-mediated cellular effects. Photosensitizer (PS) localizes in tumor cells and is converted from its ground to singlet state form through light activation. Singlet state PS can lose energy in form of heat or fluorescence, but an ideal photodynamic PS undergoes inter-system crossing and transforms into the triplet state form. Triplet state PSs with a long lifespan mediate reactive oxygen species (ROS) generation within cells. ROS induce cytotoxic effects (predominantly apoptotic and necrotic types of responses, with the exception of autophagy, which is more cytoprotecting than cytodamaging), causing cell damage and destruction.

Photochemotherapy of cancer, also known as photodynamic therapy (PDT), is a sequential photochemical and photobiological process that aims to irreversibly target and damage malignant tissues [44]. This therapy is an evolving and clinically-approved approach that exerts selective cytotoxic activities on malignant cells. It involves the administration of a photosensitizer (PS), followed by local irradiation at a wavelength that matches the absorbance band of the PS used [45,46,47]. In the presence of oxygen, irradiation induces activation of PS and a series of reactions leading to the generation of singlet oxygen and free radicals, followed by cell death. The mechanisms of cell death include direct tumoricidal effects, microvascular damage and induction of potent local inflammatory responses and depend mainly on the type and dose of PS used, the intensity of irradiation and the level of oxygen (Figure 1) [48,49].

In both clinical and experimental setting, most PSs do not localize in nuclei, but rather in plasma membrane, lysosomes, mitochondria, endoplasmic reticulum (ER) and Golgi apparatus. PSs enter cells through the plasma membrane or by endocytosis, and this subcellular localization depends on the physico-chemical properties of PSs [50,51]. For optimal photodynamic actions and efficiency, the PSs used should have an absorption peak between 600 and 800 nm, as at longer wavelengths than 800 nm, photons become inefficient and generate insufficient energy to excite oxygen to its singlet state and therefore yield insufficient ROS and cell damage. Light that penetrates into tissues is directly proportional to the wavelength. PSs with relatively strong absorbance in the red or near-infrared region of the electromagnetic spectrum are often preferred over the blue light-absorbing PSs, as blue light penetrates less effectively through tissues than red light [48]. Good PSs have limited dark toxicity and relatively rapid clearance from normal tissue, thus reducing phototoxic side effects. Photodynamic actions are sometimes associated with an acute inflammatory response characterized by an increased level of inflammatory cytokines and accumulation of leukocytes in targeted tumor areas, and PDT-mediated anti-tumor immunity has been reported [52,53]. All of these lead to an acute stress response that includes changes in the Ca^2+^ level, lipid metabolism, cytokine production and stress mediators [11]. In many photodynamic responses, the inductor stimulus is ^1^O_2_, originating from mitochondria, which enhances the formation of the receptor interacting protein 3 (RIP-3) complex. The cellular mechanism by which ROS generate such a response is not well understood [54,55].

**Figure 2:**
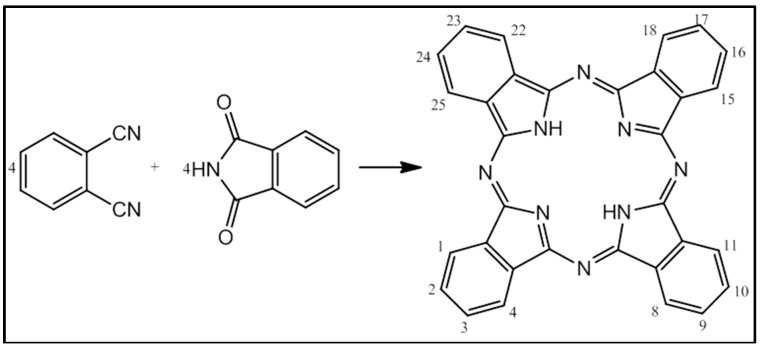
Synthesis of phthalocyanine (PC). The *O*-cyanobenzamide reacts with phthalimide to give a tetrabenzoporphyrin, also known as phthalocyanine. The structure of PC resembles that of the precursor porphyrin. PCs are tetradentate and capable of coordinating metal ions within their nitrogenous core. Chemical alternations are made possible at the metal center and substitutions at the benzo periphery (Portions 1–4, 8–11, 15–18 and 22–25).

Porphyrin had attracted the attention of photodynamic researchers and has good planar aromatic ring structures and photophysical properties with synthetic adaptability [56]. Due to the poor relative yield and limited efficiency in photodynamic applications, porphyrin is subjected to chemical modifications in order to improve its photochemical features and photodynamic therapeutic effectiveness, from the first generation into its second, then third generation. Phthalocyanine evolved from porphyrins and shares some of features of its precursors (Figure 2). Moreover, it is a better photodynamic agent with a higher yield and improved spectroscopic properties that are within the therapeutic window. Those synthetic modifications increase also the specificity of PC for neoplastic targets [53,57].

Phthalocyanine-mediated PDT has been shown to enhance autophagy, caspase-3 activation and increased cell killing [58]. After their unintended discovery during the preparation of phthalimide from phthalic anhydride in 1928, phthalocyanines (PCs) and their derivatives have since attracted attention. Preliminary studies on these interesting compounds determined both their structures and names from their phthalic anhydride origin (phthalo) and deep blue coloration (cyanine). PCs are mostly red light-absorbing compounds and referred to as tetrabenzotetraazaporphyrins; macrocyclic structured compounds with isoindole and nitrogen atoms at their meso-positions [59]. These flexible and stable compounds have enhanced light absorbing abilities and have been used as chemical sensors, semiconductors, non-linear optics and photodynamic agents [60,61]. Several factors, including the degree of ligation, the nature of the central atom, the composition of the solvents, peripheral substitution, aggregation, as well as extension of conjugation, contribute to their absorbance in the optical transmission window of biological tissues [62]. In their naturally-occurring forms, the hydrophobic characteristics of their aromatic cores make them insoluble in most solvents, which ensures their durability when used as dyes. However, applications require functionalized PCs, and in order to increase the solubility in organic solvents and to reduce aggregation, different substituents are incorporated in the peripheries of PCs.

Some porphyrin-related PSs accumulate in the vasculatures of tumor cells, and subsequent irradiation induces several damages, including stasis, vascular collapse and leakage; the mechanisms of action remain unknown [44]. Metallated PCs have been identified as strong inducers of cytodamage and preferentially accumulate in tumor cells, where they stimulate photodamages in various tumor models. *In vitro*, they have been associated with the induction of the apoptotic and necrotic pathways, while *in vivo*, PCs promote cell death through translocation of activated p38 to mitochondria, phosphorylation of BCL2 and/or BCL-X2, through facilitation of cytochrome C release from mitochondria, caspase-mediated PARP cleavage and inhibition of the P13/Akt/mTOR pathway [63,64]. Most PCs adhere to the following main features: low cytotoxicity in the dark, high phototoxicity upon light activation, high selectivity and specificity for targeted tumor, rapid clearance from the body, absorption in the optical transmission window of biological tissues, high quantum yield of singlet oxygen production, solubility in water-based solutions and stability under physiological conditions [65].

Metallated PC family members were used to investigate their phototherapeutic activities in various cancer cell lines. A mixed sulfonated metallophthalocyanine with zinc as the central atom (ZnPcSmix) successfully entered cells and localized in vital organelles, including mitochondria, lysosomes and Golgi apparatus. ZnPcSmix compounds had the ability to absorb light at a wavelength of 680 nm and showed photodynamic activities, neoplastic damage and good therapeutic results in lung, colon and breast cancer cells [66,67]. The effects of the phototherapeutic activities of ZnPcSmix in lung cancer cells included a change in cell morphology, a decrease in cell viability and proliferation, an increase in cytotoxicity and further cell damage evidence. Light-activated ZnPcSmix resulted in increased ROS production in both monolayer and multicellular tumor spheroid models of lung cancer [43,68]. The mechanisms of cell death were investigated post-irradiation in breast cancer using the same PC. The abundance of apoptotic cells, degradation of nuclear materials and the increase in the level of the expression of B-cell lymphoma-2, DNA fragmentation factor alpha and caspase-2 genes concurred with the validation of apoptosis as the induced mode of cell death [67].

The effects of others PCs similar to ZnPcSmix, with the exception of the central atoms, were investigated in esophageal and breast cancer cells. Both AlPcSmix and GePcSmix were found to be effective at targeting these malignant cells and led to cytotoxicity in a dose-dependent manner. A relatively lower concentration of photoactivated AlPcSmix and GePcSmix showed greater apoptotic inducing capabilities [69]. The effects of SnPcSmix and SiPcSmix were used in esophageal cancer and compared to those of GePcSmix and two others PC mixes. All three metallated PCs led to better cancer damaging effects when compared to an unmetallated PC mix and binaphthalo-PC counterparts. GePcSmix caused and induced an inflammatory response and high intracellular ATP, which could have been an indication of a necrotic type of cell death [70].

## 2. Conclusions

It can be concluded that programmed cell death improves the quality of life by eliminating undesirable cells from organisms. The execution of this process is accomplished by different mechanisms, including cell senescence, apoptosis, autophagy and necrosis. The induction of oxidative responses and the generation of ROS are essential for these eradicating mechanisms. PDT is an efficient means to target and induce damage in cancer cells and involves PSs, which can enhance both ROS production and cancer therapy. Metallated PCs are among the best currently-used PSs *in vitro*, and their use in clinical settings should be encouraged for prospective means of managing cancer.
